# Advancement of Nanofibrous Mats and Common Useful Drug Delivery Applications

**DOI:** 10.1155/2022/9073837

**Published:** 2022-04-19

**Authors:** Hamza Abu Owida, Jamal I. Al-Nabulsi, Feras Alnaimat, Ashraf Al Sharah, Muhammad Al-Ayyad, Nidal M. Turab, Mustafa Abdullah

**Affiliations:** ^1^Medical Engineering Department, Faculty of Engineering, Al-Ahliyya Amman University, Amman 19328, Jordan; ^2^Computer Engineering, Faculty of Engineering, Al-Ahliyya Amman University, Amman 19328, Jordan; ^3^Department of Networks and Information Security, Faculty of Information Technology, Al-Ahliyya Amman University, Amman 19328, Jordan; ^4^Civil Engineering, Faculty of Engineering, Al-Ahliyya Amman University, Amman 19328, Jordan

## Abstract

Electrospinning enables simple and cost-effective production of polymer nanofibers from different polymer materials. Drug delivery systems are capable of achieving maximum drug treatment benefits by significantly reducing adverse complications. Electrospun nanofibers have recently attracted considerable attention owing to their distinctive properties, including flexibility and biocompatibility. The implementation of functional constituents within nanostructure fibers blends is an effective technique for the administration of a variety of drugs in animal research, broadening the nanofiber capability and reliability. The nanofibrous mesh and its various application purposes are discussed in terms of a summary of recent research, emphasizing the ease of streaming and a large number of combinations of this approach, which could lead to a breakthrough in targeted therapy.

## 1. Introduction

Pharmaceutical research on innovative drugs is one of the largest challenges in both academia and industry [1]. In 2019, pharmaceutical industries worldwide were estimated to have spent 83 billion dollars on the research and development of new pharmaceuticals [2]. Most drug candidates fail at the stage of clinical trials, due to unexpected toxicity or inadequate effectiveness to treat the targeted medical issue [3]. The delivery method has a significant impact on a drug's therapeutic value, as scientists have discovered in the past decades [4].

**Figure 1:**
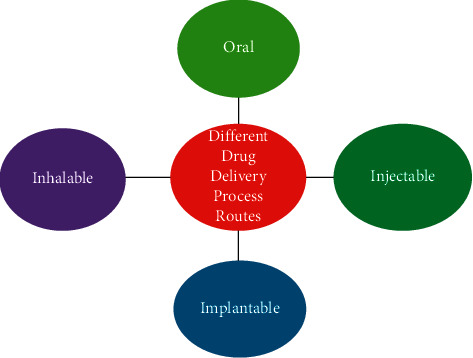
Different drug delivery process routes.

Drug efficacy can be greatly affected by how it is delivered. To better control the pharmacokinetics, pharmacodynamics, toxicity, and efficacy of drugs, new drug delivery systems have been developed. Multiple routes of administration are available for the administration of drugs to the human body [5, 6] (Figure 1).

The advancement in pharmaceutical drug delivery system is related significantly to drug manufacturing process. An effective drug delivery system depends heavily on pharmaceutical excipients. Excipients, by and large, have no medicinal value. This tool's primary function is to make the drug product production process more efficient and, as a result, to improve the drug's physiological absorption. It is possible that excipients could help with lubrication, flowability, and disintegration, as well as taste and antimicrobial properties. A critical step in the production of pharmaceutical formulations is the selection of an appropriate excipient. According to the intended use, the excipients are classified in various functional classifications as binders, dilutes, disintegrants, lubricant, wetting agents, solvents, fillers, emulsifier, absorption enhancer, and matrix for sustained release, preservatives, sweeteners and stabilizers, and coloring or flavoring. For example, gelatin and alginate were often found to be structurally simple and biologically inert biocompatible natural excipients [7–10].

The practice of drug administration has evolved dramatically over the last few decades. In the field of drug delivery, the ultimate goal is to exclusively direct therapeutic agents to pathological tissues in order to increase therapeutic efficacy and eliminate side effects [10, 11]. Researchers understand more about the different ways our bodies respond to illness and the impact of specific environmental or genetic cues as they study how diseases develop and progress. This increased understanding, when combined with technological advancements, suggests new approaches for drug delivery research [12, 13]. Current drug delivery system research can be divided into four broad categories: delivery routes, delivery vehicles, cargo, and targeting strategies.

Novel nanotechnology-based drug delivery approaches have emerged as new and exciting tools in the pharmaceutical industry [14, 15]. One of the most promising uses of this nanotechnology is in the delivery of drugs [16]. Electrospinning is an attractive drug delivery technology owing to its high loading capacity, high encapsulation efficiency, ability to deliver a variety of therapies simultaneously, simplicity of operation, and low cost. Electrospun fibers as drug carriers have a promising future in biomedical applications [17, 18]. Over the years, electrospinning was demonstrated as one of the most labored, convenient, and versatile fabrication tools for nanostructure fiber fabrication [16]. The use of nanofibers fabricated with biodegradable and biocompatible polymer expanded owing to their versatility, efficiency, and distinctive physical and chemical characteristics, such as a porous structure and a high surface area with an ultrafine diameter [16, 19]. Electrospun nanofibers for medical applications are an ideal platform for the development of a diverse range of biomedical innovations and hold great promise in the development of new and exciting products for the treatment of disease and injury [20, 21]. Hence, they have some of the most exciting applications in the biomedical fields, including tissue engineering and regenerative medicine, implant coatings, and drug delivery [20]. In the field of tissue engineering and regenerative medicine, electrospun nanofibers are used to fabricate materials and structures that closely mimic the native extracellular matrix of body tissues and optimize conditions for cell regrowth and tissue repair [19]. Electrospinning permits the use of a wide variety of materials, allowing the scaffold material's properties to be tailored to the needs of a particular application, including different biosorption times, bioactivity, and mechanical properties [22]. Smart coatings for medical devices such as stents, heart valves, and bone implants are another area where electrospinning devices for medical applications offer significant advantages over more traditional coating techniques [23, 24]. Nanofibrous scaffolds can also be used for in situ drug delivery, minimizing negative side effects of structural conventional administration of free drugs or other drug administration techniques, while increasing pharmaceutical drug activity by a slow and steady release at the action site [25]. Electrospun fibers are incredibly beneficial at integrating biological molecules into their structure [14]. Devices with higher levels of sophistication are capable of delivering various drugs with bioactivity or tuning the discharge of the encapsulated drug in reaction to a stimulus [16]. Electrospinning for drug delivery is discussed to provide a better understanding of different approaches and field applications.

## 2. Electrospinning Setups and Process

**Figure 2:**
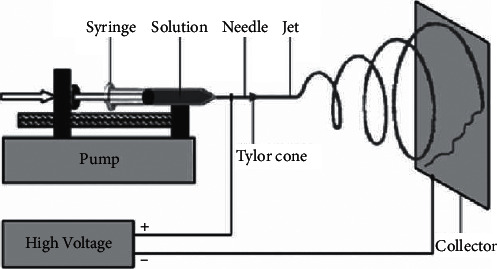
Schematic of general electrospinning setup.

Electrospinning generates nanostructured fibers by utilizing a high-voltage electrostatic potential.  Figure 2 illustrates a schematic of the electrospinning setup, which comprises three main parts: a high-voltage supply, a syringe pump, and a collector [26].

The electrospinning process is based on the use of a strong electric field. A syringe pump delivers the polymer solution to the device. The high voltage electrifies the droplet of polymer solution, and the induced charges are distributed equally above the surface. This conical object is referred to as a Taylor cone, and it results in the electrostatic field deforming the liquid droplet. Once the voltage reaches a certain level, the electric force overcomes the droplet's surface tension, causing one or more charged jets of solvent to be repelled from the droplet tip. The solvent evaporates as the jet approaches a collecting metal screen (counter electrode), and a nonwoven fabric mat forms on the screen [16, 18, 21]. Process parameters, such as the applied electric field strength, flow rate, solution concentration, viscosity and conductivity, surface tension, the distance between the syringe and collector, and ambient parameters, all have an impact on the resulting morphology of electrospun fibers [16, 25].  Table 1 illustrates the effects of electrospinning parameters on morphology of electrospun fibers.

## 3. Developed Electrospinning Setup

The basic setup of electrospinning is simple, and it has already been established in numerous research laboratories. The majority of systems were primarily designed for electrospinning from a single polymer solution or melt [16]. The basic setup, however, cannot electrospun a variety of polymers into fibers. Numerous efforts have been made to modify electrospinning equipment to broaden its universality and tailor the structure of the resulting fibers [24, 27]. Gupta and his colleagues created an electrospinning device that simultaneously electrospun two polymer solutions side by side. In this case, the two polymer solutions do not come into physical contact until they reach the end of the spinneret, where the fiber formation process begins. This side-by-side approach results in bicomponent fibers with properties from each of the polymeric components. The viscosity and conductivity of each polymer solution are critical process parameters for bicomponent electrospinning [28]. Coaxial electrospinning, like conventional electrospinning, uses a coaxial sprayer with two different-sized spinners, one of which wraps around the other [29]. The core polymeric spray is shuttled by an inner diameter smaller than the larger one, while the shell solution is transported by the nozzle with a bigger interior diameter. From the case and the core polymeric spray, pumped simultaneously from two distinct storage tanks, the core-shell nanofiber and the spinner are generated using the identical process of the equally conventional electrospinning via the voltage differential [30–32]. Because only the shell polymeric spray must be electrospun in coaxial electrospinning, the electrospun biopolymers can implement nonelectrospun drugs and growth factors into their core solution [32].

## 4. Kinetic Release Profile

Drugs can be implemented into the fiber using various methods, including immediate mixing of the drug and the polymeric mixture, surface absorption after the fabrication route, and the use of emulsification [33]. Numerous carrier materials, including natural and synthetic polymers and a blend of both, have been studied for electrospinning [21]. Furthermore, the drug release behavior is determined by drug diffusion and degradation of the carrier polymer. Electrospinning methods are used to manage the drug's distribution condition in the fibers, thereby improving drug release kinetics [34]. Understanding the release kinetics enables fine-tuning of the desired behavior by selecting the best fiber fabrication technique. The release profiles are strongly affected by multiple production processes, fiber morphology, and drug loading [35]. Li et al. in 2020 formed a multilayer synthesis by mixing multiple drugs within various polymers to treat breast tumors [36]. Researchers successfully achieved discharge on a predetermined date and time of a variety of complementary chemotherapeutics. In this example, drug discharge is strenuously affected by the rate of implementation of drugs within such a polymer structure and the drug's preference for polymeric materials [36]. In 2020, Wu et al. suggested a potential by analyzing the characteristics of poly(D,L-lactide-co-glycolide) merges including ciprofloxacin [37]. The kinetics of release prompted the recognition of three distinct phases: throughout the initial few hours, discharging occurred via phase one, defined as a first-order formula describing the distribution where the integrated substance is monitored by the lump of the fibers [37]. The second phase, lasting several days, seemed to have a more shapely and long-lasting release, as indicated by the Higuchi model's zero-order equation. The last stage was defined primarily by hydrolysis of scaffold-derived small oligosaccharides, whose distribution within encapsulated substances regulates the drug discharge ratio. It is a stage that progresses with time and lasts until the full discharge of the fiber. The disadvantages of burst release could be mitigated by twisted pair electrospinning, creating a shelled nanofibrous mesh [37]. The polymeric centerpiece is typically a barrier layer between the center and the mixture and as a drug-integrated barrier. The existence of the shield in a central nan-mesh enables prolonged release, thereby effectively preserving the drug from environmental degradation [38]. Another study created Ca-alginate microspheres loaded with bovine serum albumin before poly(L-lactic acid) nanofibrous fabrication without dispersions [39]. The results demonstrate not only effective bovine serum albumin integration but also having a 12x longer maintained actual release time compared to free microspheres.

The surface area to volume ratio is a significant indicator of the efficiency of most drug delivery systems. Nanofibrous mat-based drug systems exhibit improved solubility and bioavailability due to the small size and large surface area of nanofibers [40]. Because of this, the surface area to volume ratio plays a crucial role in the drug release kinetics, which is a key factor as it speeds up the transfer rate processes and increases interaction sites surfaces. This leads to increased release of the loaded medication. High surface-area-to-volume ratios, high porosity, and the possibility of controlling the drug release transition in nanofibers can improve drug dissolution behavior for small molecules with poor solubility [41]. The term “mucoadhesion” refers to the bond formed by two substances sticking together, at least one of which has a mucosal surface. Mucosal drug delivery has received significant attention in the last few decades [41]. Several electrospun nanofibers have mucoadhesive properties, allowing for strategic application in various mucosal tissues as a controlled delivery system for specific pharmaceutical drugs to treat a variety of pathologies. One of the many intriguing properties of nanofibers is their high encapsulation efficiency and flexible encapsulation capacity [42]. Furthermore, due to the intimacy and duration of contact, the mucoadhesion property is used to temporarily immobilize a delivery device on a specific site for targeted release and optimal drug delivery. Furthermore, the fabrication of mucoadhesive nanofibers allows for the control of the drug delivery rate via fiber degradation or drug diffusion from core-shell nanofibers, providing flexibility to position it in any part of the mucosa. The tridimensional scaffolds create a higher surface area and more contact points between the system and mucosa [43, 44]. Brako et al. prepared a mucoadhesive of carboxymethyl cellulose fibers in various concentrations of the polymer, loaded with progesterone. These mats showed about 10 times better adhesion with an artificial cellulose acetate membrane compared to that of lamb esophageal mucosa, demonstrating that carboxymethyl cellulose affects the roughness of the fibers and enhances interpenetration, improving its mucoadhesion [45]. Malik et al. proposed a mucoadhesive prepared with poly(L-lactic acid) nanofibers loaded with diacerein. The objective of this study was to describe the ability of nanofibers as a gastroretentive dosage forming the capacity to improve the solubility of diacerein. These nanofibers were smooth, discrete, and nonwoven and demonstrated a 61.3% drug release in ∼30 h [46]. Grewal et al. developed a transmucosal mucoadhesive composed of poly(e-caprolactone) nanofibers loaded with diclofenac sodium for analgesic and anti-inflammatory purposes. These fibers were characterized in terms of their *in vitro* release using a Franz diffusion cell. The fibers improved therapeutic efficacy compared to a standard method of administration [47]. Lee et al. developed a sinonasal mucoadhesive delivery system with unstructured electrospun carrier microparticles loaded with resveratrol. The electrospun nanostructure demonstrated an improved *in vivo* residence time on site of action, as well as improved local bioavailability [48]. For possible use in the oral cavity, Pérez-González et al. developed a trilayered mucoadhesive system for the unidirectional release of dexamethasone sodium phosphate. Polyvinylpyrrolidone and poly(e-caprolactone) were used as polymeric bases and polycarbophil as an adhesion enhancer in the electrospinning process to create various layers. For a four-hour release period, *in vitro* studies showed a drug content release of 79.86% of the loaded drug. In terms of mucosal adhesion, these test results are outstanding. Because of excellent thermal and mucoadhesive properties, the electrospun mucoadhesive patch demonstrated excellent stability and released an adequate amount of dexamethasone sodium phosphate when applied to mucosal surface in a mucoadhesive system [41].

## 5. Electrospun Nanofibers for Drug Delivery Application

Several delivery systems have been studied to enhance the therapy impact by reducing the toxicity of traditional pharmaceutical formulations. In the past decade, significant focus has been placed on nanoscale preparations like polymeric micelles, complexes, and nanofibers, such as lipid nanoparticles [17]. Electrospinning provides significant flexibility in choosing materials and drugs for drug carrier applications compared to other formulations. Furthermore, this method has a large potential for loading, high efficiency for encapsulation, and simultaneous supply of multiple treatments, convenience, and low cost, all of which are appealing characteristics for use in drug delivery [17,18].

**Figure 3:**
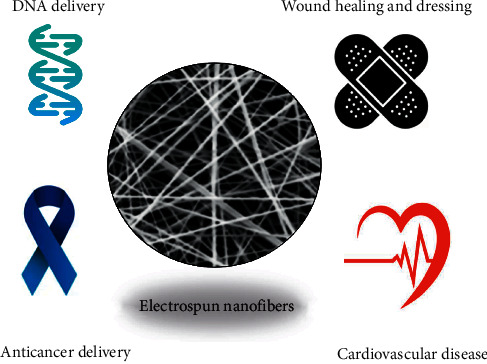
Common applications of electrospun nanofibers mat in drug delivery.

This section provides an overview of the most common and widely identified uses of nanofibers for drug delivery in the literature and the approaches for developing and characterizing these materials.  Figure 3 illustrates common applications of electrospun nanofibers mat in drug delivery.

### 5.1. Wound Healing and Dressing Electrospun Nanofibers

The skin is the largest organ of the body, with three significant roles: protection, regulatory framework, and sensory perception. As the skin serves as a boundary between the internal and external environment, its protective function makes it highly vulnerable to injury [49, 50]. Complications of chronic infections are avoided if the wound regenerates rapidly. The recovery and restoration of tissue are both intrinsically and extrinsically issues that affect wound healing [51]. Irrespective of the success of wound treatment patches and skin substitutes in the last decade, this remains a challenging task [52]. Particular requirements for wound healing nanostructures include the ability to absorb wound exudates, mimic the extracellular matrix, and be impermeable to bacteria [53]. Electrospinning is a valuable tool for achieving these characteristics. Another benefit of incorporating bioactive molecules and drugs is distribution to the sites of chronic wounds and protection against infection [54]. Jiang et al. used a PLGA/PEG-g-chitosan blend for the delivery of ibuprofen [55]. The PEG-g-chitosan component was used to mediate mechanical properties and the ibuprofen release rate from electrospun membranes. They further conjugated ibuprofen to the side chains of PEG-g-chitosan, and the drug release was prolonged for more than two weeks. Unnithan et al. introduced a new nanofibrous mat for wound tissue dressing, where a polyurethane-dextran composite nanofibrous wound dressing material was loaded with *β*-estradiol, which promotes neovascularization and skin regeneration in chronic wounds [56]. Jung et al. designed an electrospun polycaprolactone nanofiber composite with chitosan nanoparticles with fucoidan for skin wound healing. Their study demonstrated the potential for application of chitosan-polycaprolactone nanofiber composite as a wound dressing system with drug delivery for skin wound healing without side effects [57]. In 2019, Bayat and colleagues investigated the use of bromelain combined with a nanofibrous mesh of chitosan for burn wound recovery. Bromelain, a combination of proteolytic enzymes found in pineapple body tissue, was found to be acceptable for its effective debriding activity in burn injury healing [58]. In that study, fibers with good mechanical properties were produced using a simple blend electrospinning method. Moreover, only 4% of bromelain fibers were found to be cytotoxic. *In vivo* testing was performed on the scaffolds, which were contrasted in comparison to nonloaded chitosan implants. Impressive wound healing properties were demonstrated in mice, including reduced inflammation, absence of necrosis, and faster wound healing, all of which are possible with more conventional collagen fibers. In 2020, Varshosaz et al. developed the double electrospinning approach to enhance a wound dressing substrate made of customized polybutylene and gelatin nanostructures fibers combined with doxycycline [59]. Polybutylene is insoluble decomposable polyester with remarkable physical properties which is not toxic to cells. Guo and colleagues (2020) proposed a pH-dependent nanofibrous mesh for drug coloading and sequential delivery [60]. The fibers used in this study were made of a polyethylene glycol and chitosan mixture incorporated within the nanofibers was a painkiller, and polycaprolactone implanted curcumin, as well as an anti-inflammatory ingredient. The design aided in the fast release of lidocaine during the slightly earlier stages of wound healing, reducing pain instantaneously; afterward, whenever the inflammatory process started and the pH suited an environment that is significantly more acidic than biological, the discharge of curcumin was speeded up. In 2020, Faccendini et al. investigated a variety of polysaccharide-mixture-based scaffolds as skin grafts [61]. The therapies of wound infection were accomplished by loading norfloxacin, a fluoroquinolone antibiotic, on polysaccharide scaffolds. Norfloxacin was deposited as a free drug or as a montmorillonite nanocomposite into the fibers using a basic step electrospinning procedure. Scaffold drug carrier also was degraded, which occurred via lysosomes, resulting in drug release during systemic inflammation.

By creating composite nanofibers with graphene oxide, Asadi and colleagues (2020) sought to overcome zein's restricted use in wound dressings [62]. Previously, tetracycline hydrochloride was embedded within graphene oxide nanosheets. Afterwards, the distribution and mixing with the polymer solution enabled emulsification electrospinning and the formation of a composite center with the case mesh. When compared solely to zein nanofibers, graphene oxide provided improved mechanical properties and a prolonged release profile. Bakhsheshi et al. (2020) developed gentamicin combined with chitosan-alginate blended fibers [63]. Even though scaffolds revealed effective antibacterial efficiency, cell adhesion, and proliferation *in vitr*o and boosted skin regeneration in mice, the cellular metabolic analysis revealed that growth in gentamicin concentration was associated with an increase in the cytotoxic effects of the drug. Gentamicin on its own is significant in modulating the mechanical and cell adherence properties of the substrate. Hadisi et al. (2020) created hyaluronic acid and silk fibroin core-shell nanofibers [64]. Hyaluronic acid was selected because of its remarkable capacity to moderate three critical stages of wound healing: the inflammatory reaction, cell migration, and antigenicity. Even so, due to little physical effects, increased swelling, uncontrolled drug delivery, and rapid deprivation rate, it had to be combined with some other polymers. In that study, silk fibroin was chosen to address the drawbacks of hyaluronic acid while sustaining promising cytocompatibility. The nanofibers were treated with zinc oxide, which has antibacterial properties. In the *in vitro* scratch test, the nanofibrous mesh incorporated with zinc oxide demonstrated good cell adhesion and remarkable injury repair actions, as well as antibacterial properties contrary to both *E. coli and S. aureus.*

### 5.2. Antibiotics Electrospun Nanofibers Therapeutics Delivery

Infections by microbes are among the most significant and pressing problems in the medical field. Sepsis, one of the leading causes of mortality, could result from severe disease [65]. The growth of microbes can be related to antibiotic resistance. It is predicted that, by 2050, antimicrobials will be obsolete, and antibiotic resistance can lead to the deaths of 50 million people worldwide annually [66]. A microbe's capacity to pursue an antimicrobial surrounding is its antibiotic resistance; an antibiotic's inhibition concentration of antibiotic sequences is commonly employed to boost effectiveness and avoid antibiotic resistance. Furthermore, in some pathophysiological circumstances, cystic fibrosis, for example, requires a highly repetitive antibiotic process to halt the spread of chronic infections [67]. As a bioadhesive oral delivery system, Behbood et al. (2017) developed combined chitosan and gelatin fibers combined with vancomycin, a glycopeptide antibiotic [68]. In addition to improved absorption and bioavailability, these implants have a predictable release schedule and prevent hepatic first-pass metabolic activity. The rate of release of vancomycin, which has low absorption in the gastrointestinal tract and severe adverse impacts, could be an effective method to increase the dose of the drug and enhance its benefits. The nanofibrous mesh with a Fickian vancomycin release was kept constant for over three days for the *in vitro* drug release study. Wei et al. (2018) presented a drug carrier for orthopedic surgical applications, which involved the formation of polycaprolactone nanofibers to deliver vancomycin in infected critical bone defects [69].

The scaffolds' role was to aid bone healing while also controlling bacteria growth to prevent the spread of disease. Scaffolds showed outstanding biocompatibility and permitted prolonged vancomycin release for more than 14 days, with no observable release rate. In 2019, Shi et al. created a contamination-dependent nanofibrous mesh for effective targeting and anti-infection agent discharge [70]. In electrospinning, fibers coated with polydopamine permitted a larger amount of amino group introduction via the synthesis process with siloxanes. Metronidazole, a nitroimidazole antibiotic, has been esterified and progressively appended to the fibers' nanostructures substratum. An intelligent drug carrier can minimize microbial drug resistance by using a managed dosage of the drug *in vivo*. Boncu et al. (2020) created electrospun poly(lactic-co-glycolic acid) and polycaprolactone fibers combined with linezolid and oxazolidinone antibiotics with the sustained release which are used to prevent or treat skeletal prosthetic limb pathogens [71]. The goal was to speed up repair in the ruptured and disease-ridden nearby soft tissue and bone and reach the infection with governed linezolid release to achieve successful therapy with optimized antibiotic therapeutic dose. The electrospun meshes' efficiency decreases the necessity of double doses per day instead of a single dose, resulting in a 37-fold reduction in antimicrobial therapy compared to regular treatments. The method could help to avoid the spread of antibiotic resistance, while also allowing for more cost-effective therapies. A more thorough in *vivo* model could verify this design, potentially resulting in the development of a novel approach to long-term treatment of an infectious disease that can be established after surgical procedure. In 2020, Li and colleagues investigated the effect of an induced gastric drug carrier with prospective practical applications. *B. striata* polysaccharide, a pure galactomannan polymer, served as the base polymer for the system [72]. However, galactomannan has been used as a lyophilization wafer integrated with levofloxacin hydrochloride rather than a preliminary substantial for the electrospinning process. The tablets had a robust antibacterial initiative against *H. pylori*, which causes acute infectious gastroenteritis, with no cytotoxic activity. The wafer's high drug charging and chronic gastric retaining allows for a better treatment of *H. pylori* infection *in vitro and in vivo* compared to the free drug carrier.

### 5.3. Anticancer Electrospun Nanofibers Therapeutics Delivery

Although cancer treatment, diagnosis, and prevention have improved dramatically in recent years, it is still one of the world's most severe diseases and one of the foremost causes of global mortality [73]. Cancer is a diverse and multifaceted disease, exhibiting in clumps of cells that show unrestricted growth and can disperse throughout the body [74]. A better prognosis is usually associated with a diagnosis made in the early stages of the disease. Cancer detection at its source is essential before the disease's propagation, and the emergence of metastases could provide an entry point to chemotherapy or a surgical procedure to remove the foreign object of the solid tumor bulk, respectively [75]. Liu et al. incorporated dichloroacetate (DCA) into the polylactide (PLA) nonwoven fabrics by electrospinning. These DCA-loaded electrospun mats were directly implanted to cover the solid tumor. Results indicate that a tumor suppression degree of 96% was achieved in less than 19 days. Solid subcutaneous tumors completely disappeared from 50% of the tumor-bearing mice [76]. Chemotherapeutic drugs could be delivered locally to maintain their cytotoxic effects while minimizing the patient's overall toxic effects. As chemotherapeutic targeted delivery, electrospun scaffolds are well suited owing to their high biocompatibility and high selectivity to drug release [75]. Xie et al. fabricated cisplatin-loaded PLA/PLGA (30/70) fibers for long-term sustained delivery of cisplatin to treat C6 glioma *in vitro* [77]. The drug encapsulation efficiency was above 90%, and the cisplatin-loaded fibers showed sustained release for more than 75 days without the initial burst release. Liu et al. prepared doxorubicin (Dox) encapsulated nanofibers using PLLA as the carrier and examined its efficacy as a local chemotherapy system against secondary hepatic carcinoma [78]. Their results indicate that the majority of the loaded Dox in the fibers was released and diffused into the tumor site underneath the fiber mat, leading to a significant inhibitory effect on tumor growth and little damage to other organs. Chen et al. reported a controlled release system of titanocene dichloride by electrospun fiber and its *in vitro* antitumor activity against human lung tumor SPCA-1 cells. The titanocene dichloride released has evident inhibition effect against lung tumor cells. The system has an effect of controlled release of titanocene dichloride and may be used as an implantable anticancer drug in clinical applications [79]. Doxorubicin-releasing scaffolds were created by Kuang et al. (2018) [80]. To achieve this, the researchers used a technique known as mixture electrospinning, which involves spinning a hydrophilic polymer (polyethylene glycol) with a hydrophobic polymer (poly-L-lactic acid). Fibers made of 10% polyethylene glycol and 90% poly-L-lactic acid exhibited the intended release profile and were thus employed. Fibers made solely of polyethylene glycol fully dissolved within an hour, releasing the entire amount of drug they contained. The *in vivo* study revealed that the drug's biodistribution was restricted to the tumor's location and had no harmful effects. However, the initial doxorubicin burst may not effectively suppress tumor growth, and the antitumor impact was only minimally successful. He et al. (2019) adopted microfluidic electrospinning to create an implantable hierarchy nanostructured fiber for localized doxorubicin and lapatinib delivery [81]. Polymer micelles were first generated by self-assembling copolymers of 3-aminophenyl boronic acid-polycaprolactone (ethylene glycol), and fibers containing doxorubicin were synthesized. Second, using a glass capillary microfluidic device, a moist mixture comprised the specific micelles, glycerine, free doxorubicin, and an oil solution of poly(D,L-lactic acid). Lapatinib was monodispersed to achieve a water-in-oil mixture. An *in vivo* study confirmed the scaffolds' intriguing conduct, showing heavily regulated drug biodistribution at the cancer site and remarkable anticancer impact within a solitary embedding. After 21 days, the treated mice had a melanoma mass that was four times smaller than the control mice, and the treated rats had higher survival rates. In 2020, Zhang and colleagues developed pH-responsive nanostructures for 5-fluorouracil delivery [82]. In an early stage, the drug was covalently conjugated to keratin via a nucleophilic replacement encompassing the lethal cysteine of keratin. The polymer was blended with poly-L-lactic acid and electrospun to create a nanostructured mesh for resident cancer chemotherapy. When activated, the fibers discharge approximately 83% of the drug within the first 120 h, indicating a practical anticancer impact. In 2020, Yan and colleagues established pH-sensitive nanofibers with a core and a shell via central electrospinning. Polyvinyl chloride established the core and shell structures, respectively [83]. Doxorubicin discharge from the essential film was retained and was pH-dependent. Nanostructured fibers with the thickest shells released the lowest amount of doxorubicin. The discharge was indeed sluggish in a neutral environment, indicating a pH-dependent action. Fibers were assessed on a cell line from cervix cancer, where they only acted after three days. Thus, the morphology of the cells could not be revealed after seven days, indicating that the drug killed the cells. Aside from the intriguing conduct and low cytotoxicity, a faster discharge in the first stage may allow cancer treatments in short timeframes for breast cancer treatment. Sedghi et al. (2020) used chitosan derivative nanostructures to reduce the occurrence of local breast cancer [84]. Chitosan was first chemically improved by incorporating a tetraethyl urea thiosemicarbazone group, which increased its hydrophilicity. *In vitro*, the thiocarbonyl groups had good anticancer effects, and healthy cells were unaffected by the cytotoxicity of the substance. Furthermore, the incorporation of curcumin into the fibers, with a sustained and controlled discharge, was designed to provide better antiproliferative and antimicrobial properties of the fibers themselves.

### 5.4. Electrospun Nanofibers for Cardiovascular Disease Therapeutics Delivery

Cardiovascular disease, stroke, heart failure, and hypertension are among the world's most common killers [85, 86]. Blood pressure is highly associated with cardiovascular diseases; carvedilol can reduce high blood pressure, hence decreasing the related risks [87, 88]. However, it has low solubility in water and thus is mostly administered with other drugs to increase its absorption in the body [89]. Therefore, Potrc et al. developed an electrospun PCL nanofibers scaffold as a delivery medium for carvedilol; reports have indicated that, within four hours, up to 77% of the carvedilol was released from the PCL electrospun nanofibers; this was due to the scattering of the carvedilol into the scaffold nanocrystals form, which enabled its absorption and increased the dissolution rate of the drug [90].

Moreover, patients that suffered heart attacks undergo operations that involve the insertion of stands; however, the patient is likely to encounter archery thrombosis if not administered drugs that inhibit the formation of blood clots for a drug such as dipyridamole. Bakola et al. developed PLLA nanofibers with stent coating for dipyridamole [91]. Furthermore, the biocompatibility concerning preliminary *in vitro* studies has shown it to be a success with steady and continuous release of the drug along with the degradation of fibers. A similar prospect was carried out regarding stents, namely, self-expandable nitinol stents. Kersani et al. used chitosan and beta-cyclodextrin in the electrospun nanofibers and embedded the drug simvastatin within the nanofibers. Simvastatin is a drug usually used for the prevention of artery narrowing after the implantation of stents [92]. Moreover, drugs typically have a specific solubility that indicates their absorption and efficacy. Chitosan within the stents introducing cyclodextrin with simvastatin was shown to improve drug solubility, which was attributed to the reduction of the stent structure area that also improves loading of the drug. *In vivo* studies will demonstrate the reliability and biocompatibility of this new approach regarding the stents [92]. Another method was demonstrated by the approach by Rychter et al., using the PCL electrospun mesh and nanofibers. The authors' strategy was to use tubular structured PCL electrospun mesh and nanofibers, and, in this case, it was to prevent strokes [93]. This method proves to be promising where the load was released *in vitro* rapidly within 48 h of the delivery. Another drug that aids in stroke prevention was implemented into the PCL electrospun fibers to enhance the release of cilostazol. The authors merged the PCL with Pluronic P123 [94]; the aim was to achieve higher wettability for hydrophobic fibers. Furthermore, this wettability will help in sustaining the release of cilostazol and aid in tissue regeneration. Although the introduction of Pluronic P123 improved the wettability of the drug, it has been shown to exhibit toxicity levels depending on the amount of Pluronic P123 used compared to only using PCL fibers. Nevertheless, studies showed that using P123 with PCL fibers rather than pure PCL fibers yielded improvements and tensile properties due to studies in vitro. Moreover, regarding concentrations of the P123, it was shown that the release rate of the drug depended on its position within the polymer matrix.

### 5.5. Electrospun Nanofibers for Ocular Disease Therapeutics Delivery

Eye-related diseases, even simple irritations, are usually treated with liquid saline, commonly referred to as drops, as the eyes must constantly remain lubricated [95, 96]. The pharmaceutical industry has flooded the market with such treatment salines [97]. However, there is a more promising prospect when it comes to treatment using electrospun nanofibrous scaffolds, where the drugs and liquids can be delivered to the site of infection or irritation and can thus be more effective than conventional pharmaceutical products. In this case, the biocompatible and biodegradable scaffold infused with the drug is able to prolong the treatment with higher efficiency and reliability [98–100].

Tawfik et al. fabricated electrospun nanofibers composed of PLGA infused with pirfenidone and antifibrotic drugs. This scaffold assumed a coaxial form, which allowed it to be embedded with the mentioned drugs. This approach was to prevent bacterial infections to the eye, known as corneal abrasion [101]. Moreover, antibiotic moxifloxacin was used to treat various bacteria in the case of PVP, which had the essential property of being hydrophilic. Göttel et al. proposed a rather interesting method to deliver the drug, in which, however, the drug delivery or release rates were not completely identified. The theoretical analogy concerning the fabrication of the electrospun scaffold had promising prospects. In this case, the authors intend to fabricate a platform that would hold the treatment of topical ocular diseases and the electric spun nanofibers scaffold composed of Gellan gum/pullulan polymers [102]. The aim of using such materials is to manipulate the material into specific shapes that would cover as much surface area of the eye to increase the effectiveness of the treatment. Thereby, the delivery of the drug can be more effective and efficient than the use of the conventional pharmaceutical liquid drug.

Grimaudo et al. adopted an approach to fabricate an electrospun nanofibers scaffold containing the treatment of various types of ocular surface diseases. The authors' electrospun scaffold fabrication was composed of hyaluronan and PVP in an ophthalmic mesh to deliver ferulic acid, antioxidants, *ε*-polylysine, and antimicrobial peptides [103]. The ferulic acid was mixed with the polymer, and *ε*-polylysine was added after the electric spinning process. The results of such fabrication were claimed to be a success owing to no complications or irritations such as vessel lysis or hemorrhage and acted as the standard conventional saline drug. The drugs embedded in the scaffold were released at a continuous steady rate that took about 20 min, and the results showed high rates of biodegradability. Furthermore, the effects were noted to have high effects. *P. aeruginosa* and *S. Aureus* resulted in the inhibition of their growth; however, this method was more efficient as a short-term than a long-term treatment due to the time frame. Di Prima et al. proposed an electrospun nanofiber scaffold to deliver drugs for ocular diseases. However, the authors' approach to scaffold composition was to add acetonide-loaded poly(1,4-butylene succinate) to triamcinolone. This resulted in the highly porous electrospun nanofibers, with highly improved wettability, mucoadhesion, and cytocompatibility [104]. With these properties, drugs can be delivered efficiently with high loading rates and no irritation or complications to the corneal epithelial cells. Forouzideh et al. fabricated the electrospun nanofiber scaffold embedded with antiangiogenesis and loaded with epigallocatechin gallate [105]. The scaffold was composed of silk fibroin and treated with methanol to house more of the drug into the scaffold. The methanol improved the lipophilicity, which significantly affected the drug delivery due to the silk in the platform transforming into *β*-sheets. The platform suggested by the authors scans the drug delivery and supports cell proliferation and cell growth. Within the five days of the drug release, the scaffold made room for the growing limbal cells. Da Silva et al. worked on the drug dexamethasone, used for retinal disease treatments. PCL nanofibers loaded with acetate were employed. This approach was similar to the methods mentioned earlier; however, in this case, owing to the construction of the electrospun PCL nanofiber scaffold [106], dexamethasone had a release rate that took 12 days to complete. This duration provided time for the biocompatible PCL scaffold to be integrated during the drug release. Moreover, to ensure that no complications or irritations occurred, the fibers within the scaffold were in an acidic solvent, which ensured that no residual particle is left behind once the biodegradation is completed. Nevertheless, this duration also enabled for cell proliferation and growth to successfully occur without cytotoxicity.

### 5.6. Electrospun Nanofibers for Protein, DNA, RNA, and Growth Factors Delivery

Gene editing or impeding the mutant gene's mechanism has been demonstrated as novel approaches to treating regenerative diseases. Nevertheless, the delivery of nucleic acid molecules such as DNA or interference RNA into a target cell to knockout/knockdown mutant gene expressions was shown to be particularly useful [107, 108]. Using electrospun nanofibrous scaffolds in the treatments has proved to be highly effective. This method was also used to treat diseases related to growth factor proteins. Researchers have embedded RNA and growth factors into electrospun nanofibrous scaffolds and have shown tremendous results. In some cases, they exhibit higher effectiveness than conventional treatment [109–111]. Rujitanaroj et al. demonstrated the feasibility of delivering small interfering RNA (siRNA) and transfection reagent complexes within nanofibers comprising a copolymer of caprolactone and ethyl ethylene phosphate (PCLEEP). Coencapsulation of siRNA and transfection reagent complexes within PCLEEP fibers resulted in a sustained release of bioactive siRNA for at least 28 days [112].

Chew et al. delivered small interfering RNA using the PCL electrospun nanofibers scaffold, which proved its high efficiency in releasing small interfering RNA without complications such as toxicity and had a substantial effect on cellular transfection. However, the authors' approach proposed a different prospect with promising results, where the method implemented to the scaffold encapsulated both the B essay and the human nerve growth factor into the PCL and poly(ethyl ethylene phosphate) electrospun nanofibers [113]. Results indicate a release of the protein during three months. Furthermore, the release occurred at a continuous steady rate. Another method involved the delivery of small interfering RNA and a transfection reagent into electric by scaffolds composed of PCL and polyethylene phosphate. The scaffold showed steady degradation with good biocompatibility. Importantly, the scaffold could encapsulate the small interfering RNA within its core, which allowed a continuous delivery of the small interfering RNA with simultaneous biodegradation [114]. Geiger et al. adopted an approach of transforming growth factor-beta 1 (TGF-*β*1) into electrospun nanofibers scaffolds which were composed of alginate sulfate and polyvinyl alcohol; this method was used to deliver the transforming growth factor-beta 1, achieving successful results. The authors illustrated the results with a continuous steady release of the protein with biodegradability of the electrospun scaffold. Furthermore, the authors proposed biomimetic hydrogels with promising features in holding the proteins with a steady release, with biocompatibility and biodegradability factors. Several types of growth factors can be encapsulated within the electrospun scaffolds. The suggested scaffold is composed of poly-D-lactide nanofibers with embedded human bone marrow stromal cells and VEGF aimed to regenerate blood vessels and enhance bone growth [115]. Liu et al. adopted an approach consisting of two types of electrospun nanofibrous scaffolds. The reason was to deliver growth factors for nerve tissue engineering. The first nanofibrous scaffold was PDLLA, which housed the nerve growth hormone. The second nanofibrous scaffold was PLGA, which housed the glial cells neurotrophic factors [116]. Results showed a resulting growth that had better structural integrity than the growth factors alone.

## 6. Conclusion

Electrospinning has developed significantly over the last few decades. Electrospinning is a convenient and straightforward method of developing intelligent and controllable drug delivery systems. The potential options of electrospinning are limitless and provide an excellent base for developing innovative drug delivery applications capable of maximizing therapy value and minimizing adverse impacts. Drug and polymer selection can easily be refined for the unique use areas or provision. The nanofibrous mesh could open up novel avenues for precision medication by modifying mechanical characteristics or release kinetics. Aside from the significant advantages provided by this approach, only a few clinical studies have been documented in the literature over the years, and regulative bodies such as the FDA and EMA have yet to validate such systems. In numerous cases, the toxic distillate of the mixture cast-off produced by the spinning process remains in the fiber and is discharged within the drug.

Novel methods enabling biocompatible mixtures are far preferred to toxic ones. Melt electrospinning, which produces nanofibers without the need for any solvent, holds great potential as such a strategy. However, it is essential to shield the drug from heat and degradation. Furthermore, the rapid advancements of expertise and the development of more advanced mutual systems may aid in developing integrated intelligent devices capable of precisely modulating the amount of drug discharged from the nanofibrous substrate following body stimulation. Nanofibrous meshes in diabetes, hormonal treatment, and immune disorders are all underexplored research areas. A comprehensive and systemic methodology might aid in addressing the issues concerning electrospun nanofibers. Optimized scaffolds that combine tissue engineering with precise drug release without harmful side effects could be an effective instrument for the treatment of patients in medical facilities in the future. The distinct characteristics and ease of use of customized nanofibers could play an increasingly important role in personalized medicine.  Table 2 lists the representative drugs loaded into the electrospun mat for drug delivery.

## Figures and Tables

**Table 1:** Effects of electrospinning parameters on morphology of electrospun fibers.

Parameter	Effect of parameter on fiber morphology
Viscosity/concentration	Fiber diameters increase with increasing concentration/viscosity.
Applied voltage	Relationship between voltage and fiber diameter is difficult to ascertain.
Distance between nozzle and collector	A minimum distance is needed to acquire dry fibers. Beading is observed at either too close or too far distances.
Flow rate	Fibers with smaller diameters are produced at lower flow rates, and excessive flow rates result in fibers that are not dry upon arrival at the collector.
Solution conductivity	Higher conductivities generally result in smaller fibers, but increasing conductivity facilitates the creation of consistent bead-free fibers.
Solution additives	(i) Alcohol, the formation of beads is reduced.
	(ii) Acetone, small beads are formed.
	(iii) N,N-dimethyl formamide.
	(iv) (DMF) Bead size decreases.
Surfactant additives	(i) Cationic surfactants: the formation of beads is prevented and the proportion of the cationic surfactant is increased, resulting in thinner fibers.
	(ii) Nonionic surfactants: the number of beads decreases and the fiber morphology changes, despite the fact that bead formation is not prevented.
Ambient parameters	Upon temperature rise, the viscosity of the solution decreases, resulting in smaller fibers. Increasing humidity causes the fibers to develop circular pores.
Surface tension	Rise in the surface tension coefficient of the solutions increases the quantity of beads.

**Table 2:** Representative drugs loaded into electrospun mats for drug delivery applications.

Nanofibrous mat	Drug	Application	References
Chitosan	Bromelain	Burn wound recovery	[24]
Gelatin	Doxycycline	Skin wound dressing	[28]
Graphene oxide	Tetracycline hydrochloride	Wound dressings	[29]
Chitosan-alginate	Gentamicin	Skin wound dressing	[30]
Hyaluronic acid and silk fibroin	Zinc oxide	Wound dressings and antibacterial patch	[31]
Polycaprolactone	Vancomycin	Bone healing	[33]
Polydopamine	Metronidazole	Anti-infection agent	[34]
Polycaprolactone	Linezolid and oxazolidinone	Skeletal prosthetic limb pathogens	[35]
Galactomannan polymer	Levofloxacin hydrochloride	Antibacterial tablets	[36]
Polyethylene glycol	Doxorubicin	Anticancer	[41]
Polycaprolactone (ethylene glycol)	Doxorubicin and apatinib	Anticancer	[42]
Poly-L-lactic acid	5-FU-K-P	Anticancer	[43]
Polyvinyl chloride	Doxorubicin	Anticancer	[44]
Chitosan	Thiocarbonyl groups	Anticancer	[45]
Polycaprolactone	Carvedilol	Cardiovascular diseases	[51]
Poly-L-lactic acid	Dipyridamole	Antithrombotic	[52]
Chitosan	Simvastatin	Prevention of arteries narrowing	[53]
Polycaprolactone	Cilostazol	Prevent strokes	[54]
Polycaprolactone and Pluronic 123	Cilostazol	Prevent strokes	[55]
Poly (lactic-co-glycolic acid)	Pirfenidone	Corneal abrasion	[61]
Gellan gum/pullulan	Moxifloxacin	Topical ocular infection	[62]
Polyvinylpyrrolidone	Ferulic acid	Hemorrhage	[63]
Polycaprolactone	Interfering RNA	Interfering RNA release	[70]
Polycaprolactone and polyethylene phosphate	Interfering RNA	Interfering RNA and transfection	[71]
Alginate sulfate and polyvinyl alcohol	Transforming growth factor-beta 1	Transforming growth factor-beta 1 release	[72]
Poly (D,L-lactic acid)	Nerve growth hormone	Nerve growth hormone release	[73]

## Data Availability

The data used to support the findings of this study are included within the article.
